# Associations Between Ostomy Creation and Health‐Related Quality of Life in Colorectal Cancer Patients: A Longitudinal Observational Study

**DOI:** 10.1002/cam4.71388

**Published:** 2025-11-28

**Authors:** Jerrald Lau, Alyssa Ng, Wei‐Ling Koh, Cherie Hui Peh, The Singapore Colorectal Cancer Research Group, Bettina Lieske, Wai‐Kit Cheong, Jing‐Yu Ng, Dedrick Kok Hong Chan, Ian Jse‐Wei Tan, Kai‐Yin Lee, Bryan Buan, Jarrod Kah‐Hwee Tan, Choon‐Sheong Seow, Christopher Hang‐Liang Keh, Min‐Hoe Chew, Fung‐Joon Foo, Sharmini Su Sivarajah, Winson Jianhong Tan, Nan Luo, Ker‐Kan Tan

**Affiliations:** ^1^ Yong Loo Lin School of Medicine National University of Singapore Singapore Singapore; ^2^ Saw Swee Hock School of Public Health, National University of Singapore Singapore Singapore; ^3^ Department of Surgery National University Hospital Singapore Singapore

**Keywords:** colorectal neoplasms, health‐related quality of life, ostomy, patient‐reported outcomes

## Abstract

**Background:**

Many colorectal cancer (CRC) patients require stoma creation following surgical resection. Stomas can significantly impact health‐related quality of life (HRQOL), yet prospective longitudinal studies exploring this are limited. This study aimed to assess HRQOL among CRC patients comparing between those with and without stomas, and examining differences between subgroups.

**Methods:**

This prospective longitudinal study was conducted across five hospitals in Singapore from 2018 to 2023. CRC patients undergoing surgical resection were assessed at six timepoints from diagnosis to 12‐month post‐surgery. HRQOL was measured using the EORTC QLQ‐C30 instrument. Multilevel mixed‐effects tobit regression models were used to evaluate HRQOL changes over time and identify significant predictors of HRQOL outcomes.

**Results:**

Among 240 patients, 91 (37.9%) underwent stoma creation. Stoma patients reported significantly poorer HRQOL, particularly in global health status and functional domains at 1 month post‐surgery, which persisted into the 3‐month timepoint for role (77.43 vs. 86.67, *p* < 0.05) and emotional (82.11 vs. 90.20, *p* < 0.05) functioning. Stoma creation predicted reduced physical (*β* = −10.39, 95% CI: −15.80, −4.97) and role functioning (*β* = −12.24, 95% CI: −24.38, −0.10). Subgroup differences were mostly non‐significant, except for consistently lower cognitive functioning scores in patients with permanent as opposed to temporary stomas (e.g., 1 month; 73.08 vs. 91.19, *p* < 0.025).

**Conclusions:**

Stoma creation adversely affects HRQOL, particularly in the early postoperative period. Comprehensive post‐surgical support addressing physical, psychological, and financial challenges may be crucial to enhance overall HRQOL for stoma patients. Further research should explore tailored support strategies and the effect of early versus delayed stoma closure on HRQOL outcomes.

## 1 Introduction

Colorectal cancer (CRC) is the third highest incident cancer globally, accounting for 1.9 million of the 19.9 million new cancers in 2022 [1]. Advances in surgical techniques and oncological treatment options have resulted in improved five‐year age‐standardised survival rates to over 60.0% in both sexes across the last two decades [2]. A considerable proportion of CRC patients who undergo surgical resection will require a stoma, which typically involves the creation of either an ileostomy or colostomy and can be temporary or permanent.

It is broadly understood that undergoing an ostomy can affect a CRC patient's health‐related quality of life (HRQOL), which is a key patient‐reported indicator that has also been associated with clinical outcomes. Studies have demonstrated that higher HRQOL scores are predictive of overall survival in cancer patients, especially in domains such as overall health status and physical functioning [3, 4, 5]. A recent narrative systematic review by Alenezi and colleagues highlighted that stoma patients potentially suffer from a range of challenges that negatively impact HRQOL [6]. These include physical (e.g., increased fatigue, insomnia), psychological, and social issues (e.g., poor self‐image, depressive feelings, self‐isolation), and can also include difficulties performing activities of daily living and poor self‐care [6, 7].

The lack of prospective longitudinal studies is a gap in the literature on HRQOL of cancer patients who undergo ostomy creation. In Vonk‐Klaassen and colleagues' systematic review, for example, 12 of the 14 observational studies that were included utilized a cross‐sectional approach [8]. Similarly, more recent studies investigating HRQOL in stoma patients over the last five years have continued to predominantly use cross‐sectional designs [9, 10, 11, 12]. While these provide an important snapshot of the post‐operative HRQOL experience of stoma patients, cross‐sectional studies preclude evaluations of how ostomy creation may contribute to changes in a CRC patient's HRQOL over the course of their treatment journey, which can be complicated by other factors such as chemotherapy.

The objective of the present research was therefore to describe HRQOL in CRC patients who underwent ostomy creation from diagnosis to 12 months post‐surgery in a multi‐ethnic Southeast Asian population. We also sought to compare these HRQOL trends between CRC patients with stoma versus those without and examine if HRQOL differences exist between different subgroups of stoma patients (i.e., temporary vs. permanent, ileostomy vs. colostomy).

## 2 Methods

### 2.1 Study Design

This was part of a prospective longitudinal observational study designed to assess HRQOL in CRC patients over a 12‐month period following curative surgery. Five public hospitals in Singapore served as study sites—National University Hospital (NUH), Ng Teng Fong General Hospital (NTFGH), Sengkang General Hospital (SKGH), Changi General Hospital (CGH) and Khoo Teck Puat Hospital (KTPH). Participants were recruited between February 2018 and January 2023. Recruitment efforts were disrupted from February 2020 to December 2021 due to COVID‐19 pandemic restrictions in Singapore, which imposed a moratorium on non‐essential research activities.

### 2.2 Participants

Patients were recruited from each site's respective colorectal outpatient clinics. They were eligible to participate if they had a confirmed histological diagnosis of CRC scheduled for surgical resection in one of the study sites. Patients with concurrent malignancies were excluded. Informed written consent was obtained from all participants prior to enrolment in the study. Each participant completed a single questionnaire over six timepoints: Baseline (point of diagnosis), and at 1, 3, 6, 9, and 12 months after surgery. Each questionnaire was administered either in person at the outpatient clinics or via telephone by the study team.

### 2.3 Ethics Approval

Ethical approval for the study was obtained from the National Healthcare Group's Domain Specific Review Board (NHG DSRB Ref: 2017/00518). All patients gave their informed consent prior to their inclusion in the study.

### 2.4 Measures

The questionnaire consisted of the European Organisation for Research and Treatment of Cancer Core Quality of Life Questionnaire (EORTC QLQ‐C30). The EORTC QLQ‐C30 is a widely validated patient‐reported outcomes instrument used in oncology research. The 30‐item questionnaire evaluates overall HRQOL across several domains, consisting of a global health status (GQOL) scale, five functioning scales (physical, role, emotional, cognitive, and social), three symptom scales (fatigue, nausea/vomiting, and pain), and six single‐item measures (dyspnoea, insomnia, appetite loss, constipation, diarrhoea, and financial difficulties) [13]. Psychometrically, the EORTC QLQ‐C30 has been shown to have good content validity and moderately high internal consistency, with alpha values ranging from 0.62 to 0.87 [14, 15]. Each question was answered on a 4‐point scale ranging from “Not at all” to “Very much” except GQOL, which used a 7‐point scale. Responses were linearly transformed into composite scores for each domain ranging from 0 to 100 based on the EORTC's scoring manual [16]. Higher scores on the GQOL and functioning scales reflect better functioning while higher scores on the symptom scales indicate more severe symptoms. The constipation and diarrhoea measures were omitted from the analysis as these were irrelevant to patients with stoma and hence incomparable between groups.

Musuro and colleagues' work establishing minimal important differences (MID) for the EORTC QLQ‐C30 was used to provide additional nuance [17]. These scoring guidelines help define the magnitude of improvements or deteriorations for each EORTC QLQ‐C30 domain between groups or measurement timepoints. For example, a global health status mean score difference of between −4 and 6 points is considered marginal and unlikely to result in any noticeable difference in the patient's actual HRQOL in a clinical setting, even if the analysis was statistically significant.

Demographic and clinical data were extracted from patients' electronic medical records using a standardised data collection form. These comprised age, gender, marital status, tumour site (rectal/colon), cancer staging, pre‐operative morbidity (using American Society of Anesthesiologists classification; ASA), mode of surgery (laparoscopic/open), post‐operative adjuvant treatment (i.e., chemotherapy, radiotherapy), whether the patient underwent ostomy, type of stoma created (ileostomy/colostomy), whether the stoma was temporary or permanent, post‐operative length of hospital stay (LOS), 30‐day readmission (yes/no), and severity of post‐operative complications (using Comprehensive Classification Index; CCI).

### 2.5 Statistical Analyses

Statistical analyses were conducted using Stata/BE 17.0 (StataCorp LLC). All available data was used, and no missing data imputation was performed. Missing data was calculated as the proportion of completed outcomes per timepoint over the initial baseline sample size. Descriptive statistics were used to summarize baseline characteristics. Demographic statistics were presented using means (with standard deviation) or frequencies (with proportions) for continuous and categorical variables respectively. Bivariate baseline analyses between the stoma and non‐stoma groups were performed using chi‐square test or Fisher's exact test for categorical variables and independent samples t‐test for continuous variables, with *p* < 0.05 denoting statistical significance.

To assess changes in HRQOL outcome scores over time, multilevel mixed effects tobit regression models were used with *p* < 0.05 denoting statistical significance. Multilevel mixed effect tobit regressions are similar to linear mixed models in that they allow the modelling of relationships between fixed and random effects on repeated measures (e.g., HRQOL scores over multiple timepoints). However, tobit models additionally account for potential ceiling effects, which in our sample were observed by the high mean scores and standard deviations in multiple HRQOL domains (e.g., physical, emotional and social functioning) that were close to the maximum possible score of 100. As demographic and clinical data were extracted based on patient characteristics routinely collected and available to clinicians within the participating institutions, potential fixed effect covariates were identified via a data‐driven approach—from baseline characteristics that were significantly different between groups. This was performed to ensure that the fixed effect covariates identified would be generalisable to our broader source population. These covariates were entered into each model using a forward stepwise process, along with classical demographic factors (age, marital status, gender). Covariates were only retained if their inclusion improved the model fit, per the Akaike and Bayesian information criteria (AIC, BIC). The outcomes of interest were scores from the EORTC QLQ‐C30's functioning scales and single‐item measures. Each model also included the baseline score of the respective outcome as a fixed effect covariate. Random effects were included to account for variability in HRQOL trajectories between patients across the study time points. Due to the low number of patients with permanent stomas, stoma permanence was not used as a covariate. Instead, a sensitivity analysis was performed by running the multilevel mixed effects tobit regression models with permanent stoma patients excluded from the analysis.

Descriptive and bivariate subgroup analyses were additionally performed to examine differences in baseline characteristics and HRQOL scores (at 1‐, 3‐, 6‐, 9‐, and 12‐month timepoints) between (A) patients who underwent ileostomy versus colostomy and (B) patients who had temporary versus permanent stomas. Mann–Whitney *U* test was used for bivariate comparisons involving these subgroups. Longitudinal analyses were not performed owing to the relatively small subgroup sample sizes. To account for the possibility of inflated Type I error, Bonferroni correction for multiple comparisons was used in all bivariate subgroup analyses, with an adjusted *p* < 0.025 denoting statistical significance.

## 3 Results

### 3.1 Baseline Characteristics

A total of 240 patients were included in this study, of which 91 (37.9%) underwent ostomy. The stoma group had significantly more rectal cancers (*N* = 58; 63.7%), more patients who underwent open instead of laparoscopic surgery (*N* = 21; 28.4%), and radiotherapy (*N* = 32; 36.0%) respectively. The stoma group also had significantly higher CCI (mean score = 11.23) and LOS (mean days = 11.05). A comparison of baseline demographic and clinical characteristics can be found in Table S1.

### 3.2 EORTC QLQ‐C30 Scores Between Patients With and Without Stoma

**FIGURE 1:**
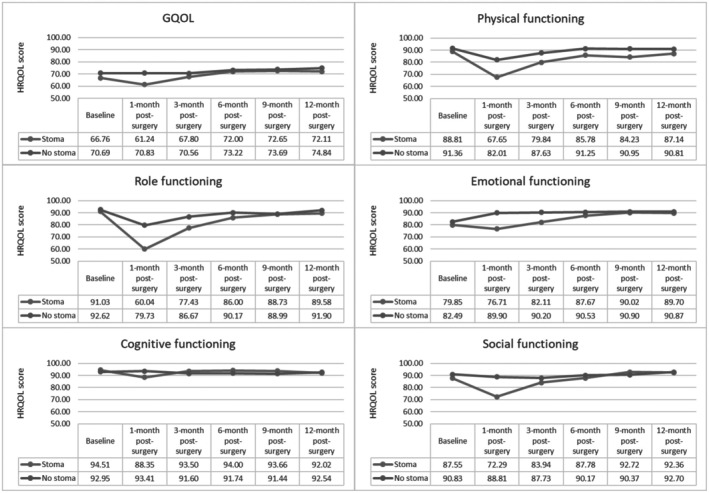
Mean scores for GQOL and functional scales between the stoma and no stoma groups across the study period.

**FIGURE 2:**
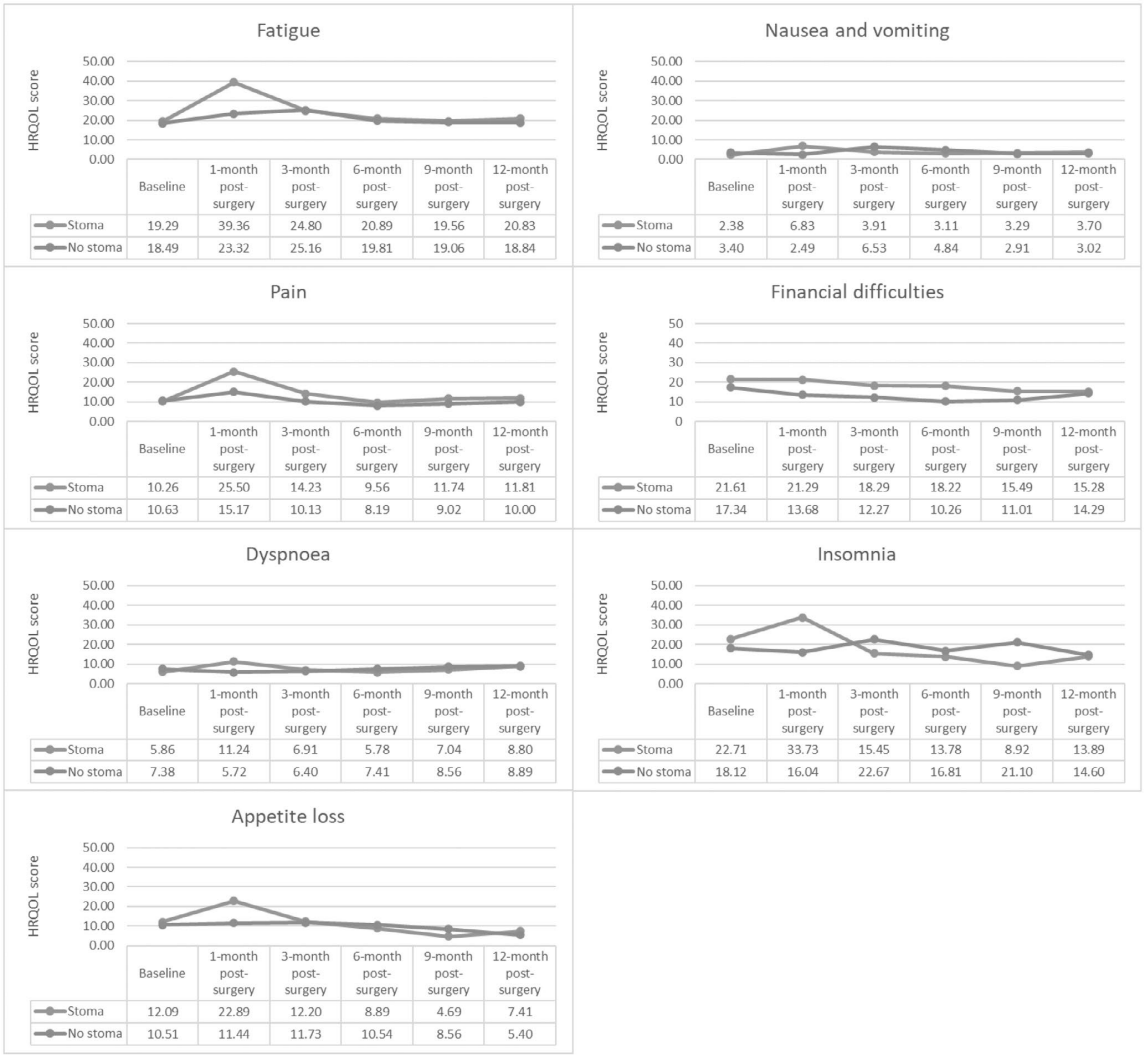
Mean scores for symptom scales between the stoma and no stoma groups across the study period.

Figures 1 and 2, together with Table 1, present graphical trends as well as bivariate comparisons respectively at each timepoint of the EORTC QLQ‐C30 scores between stoma and no stoma groups. The stoma group consistently experienced clinically and statistically significantly poorer HRQOL across the GQOL and functional scales at the 1‐month post‐surgery timepoint, except for cognitive functioning (88.35 vs. 93.41, *p* = 0.06). Statistically, this persisted into the 3‐month timepoint for role (77.43 vs. 86.67, *p* < 0.05) and emotional (82.11 vs. 90.20, *p* < 0.05) functioning although MIDs were unavailable for the emotional functioning domain. Physical functioning was also significantly poorer both statistically and clinically in the stoma group at all post‐surgery timepoints except the 12‐month, where the difference became minimal (approximately 3.00 points).

**TABLE 1:** EORTC QLQ‐C30 scores between stoma and no stoma groups across the study period.

GQOL and functioning scales, mean (SD)	Stoma (*N* = 91)	No stoma (*N* = 149)	*p*	Missing data (%)
*Global health status*				
Baseline	66.76 (20.24)	70.69 (19.80)	0.14	—
1‐month post‐surgery	61.24 (20.10)	70.83 (16.08)	< 0.05*	10.00
3‐month post‐surgery	67.80 (19.44)	70.56 (17.25)	0.30	15.00
6‐month post‐surgery	72.00 (20.07)	73.22 (17.84)	0.67	20.42
9‐month post‐surgery	72.65 (16.26)	73.69 (17.88)	0.69	26.25
12‐month post‐surgery	72.11 (18.14)	74.84 (16.90)	0.31	27.08
*Physical functioning*				
Baseline	88.81 (18.37)	91.36 (14.49)	0.26	—
1‐month post‐surgery	67.65 (25.52)	82.01 (17.45)	< 0.05*	11.25
3‐month post‐surgery	79.84 (22.42)	87.63 (16.41)	< 0.05*	14.17
6‐month post‐surgery	85.78 (18.99)	91.25 (12.44)	< 0.05*	21.25
9‐month post‐surgery	84.23 (20.13)	90.95 (13.77)	< 0.05*	25.83
12‐month post‐surgery	87.14 (18.22)	90.81 (15.48)	0.17	28.33
*Role functioning*				
Baseline	91.03 (19.45)	92.62 (18.82)	0.53	—
1‐month post‐surgery	60.04 (34.53)	79.73 (26.83)	< 0.05*	11.25
3‐month post‐surgery	77.43 (30.36)	86.67 (23.57)	< 0.05*	14.17
6‐month post‐surgery	86.00 (23.74)	90.17 (18.97)	0.20	21.25
9‐month post‐surgery	88.73 (21.96)	88.99 (20.06)	0.94	25.83
12‐month post‐surgery	89.58 (20.64)	91.90 (18.93)	0.45	28.33
*Emotional functioning*				
Baseline	79.85 (18.60)	82.49 (20.24)	0.30	—
1‐month post‐surgery	76.71 (25.86)	89.90 (16.15)	< 0.05*	10.00
3‐month post‐surgery	82.11 (21.08)	90.20 (16.05)	< 0.05*	14.17
6‐month post‐surgery	87.67 (18.19)	90.53 (14.92)	0.26	20.42
9‐month post‐surgery	90.02 (15.01)	90.90 (15.24)	0.70	25.83
12‐month post‐surgery	89.70 (13.45)	90.87 (15.61)	0.59	27.08
*Cognitive functioning*				
Baseline	94.51 (11.40)	92.95 (12.58)	0.33	—
1‐month post‐surgery	88.35 (20.62)	93.41 (15.57)	0.06	10.00
3‐month post‐surgery	93.50 (12.76)	91.60 (15.50)	0.34	14.17
6‐month post‐surgery	94.00 (12.45)	91.74 (13.95)	0.25	20.42
9‐month post‐surgery	93.66 (13.32)	91.44 (16.45)	0.32	25.83
12‐month post‐surgery	92.02 (14.05)	92.54 (14.06)	0.81	27.50
*Social functioning*				
Baseline	87.55 (18.32)	90.83 (18.32)	0.24	—
1‐month post‐surgery	72.29 (33.77)	88.81 (20.62)	< 0.05*	10.00
3‐month post‐surgery	83.94 (24.91)	87.73 (22.81)	0.27	14.17
6‐month post‐surgery	87.78 (21.11)	90.17 (19.71)	0.43	20.42
9‐month post‐surgery	92.72 (16.60)	90.37 (20.57)	0.40	25.83
12‐month post‐surgery	92.36 (19.16)	92.70 (16.00)	0.90	27.08

Symptom scales (excluding constipation and diarrhoea), mean (SD)	Stoma (*N* = 91)	No stoma (*N* = 149)	*p*	Missing data (%)
*Fatigue*				
Baseline	19.29 (19.16)	18.49 (19.78)	0.76	—
1‐month post‐surgery	39.36 (27.67)	23.32 (20.68)	< 0.05*	10.83
3‐month post‐surgery	24.80 (21.83)	25.16 (25.05)	0.91	14.17
6‐month post‐surgery	20.89 (20.09)	19.81 (21.57)	0.72	21.25
9‐month post‐surgery	19.56 (18.45)	19.06 (22.18)	0.87	25.83
12‐month post‐surgery	20.83 (20.83)	18.84 (20.16)	0.53	27.08
*Nausea and vomiting*				
Baseline	2.38 (11.80)	3.40 (9.54)	0.49	—
1‐month post‐surgery	6.83 (17.47)	2.49 (8.79)	< 0.05*	10.00
3‐month post‐surgery	3.91 (9.60)	6.53 (14.02)	0.11	14.58
6‐month post‐surgery	3.11 (10.14)	4.84 (12.58)	0.30	20.42
9‐month post‐surgery	3.29 (14.81)	2.91 (7.81)	0.84	25.83
12‐month post‐surgery	3.70 (14.05)	3.02 (10.79)	0.73	27.08
*Pain*				
Baseline	10.26 (20.30)	10.63 (16.12)	0.88	—
1‐month post‐surgery	25.50 (27.59)	15.17 (20.07)	< 0.05*	10.00
3‐month post‐surgery	14.23 (21.46)	10.13 (17.44)	0.15	14.17
6‐month post‐surgery	9.56 (18.62)	8.19 (15.62)	0.60	20.83
9‐month post‐surgery	11.74 (19.80)	9.02 (15.64)	0.33	25.83
12‐month post‐surgery	11.81 (19.27)	10.00 (18.86)	0.54	27.08
*Financial difficulties*				
Baseline	21.61 (33.10)	17.34 (29.48)	0.31	—
1‐month post‐surgery	21.29 (33.16)	13.68 (25.92)	0.08	10.00
3‐month post‐surgery	18.29 (29.23)	12.27 (25.24)	0.13	14.17
6‐month post‐surgery	18.22 (33.01)	10.26 (22.09)	0.07	20.42
9‐month post‐surgery	15.49 (29.19)	11.01 (22.71)	0.28	25.83
12‐month post‐surgery	15.28 (30.61)	14.29 (27.29)	0.83	27.08
*Dyspnoea*				
Baseline	5.86 (13.69)	7.38 (16.82)	0.45	—
1‐month post‐surgery	11.24 (21.00)	5.72 (12.62)	< 0.05*	10.00
3‐month post‐surgery	6.91 (17.16)	6.40 (14.48)	0.82	14.17
6‐month post‐surgery	5.78 (15.86)	7.41 (16.44)	0.49	20.42
9‐month post‐surgery	7.04 (15.85)	8.56 (18.37)	0.56	25.83
12‐month post‐surgery	8.80 (18.55)	8.89 (17.46)	0.97	27.08
*Insomnia*				
Baseline	22.71 (30.58)	18.12 (27.53)	0.24	—
1‐month post‐surgery	33.73 (35.50)	16.04 (26.46)	< 0.05*	10.42
3‐month post‐surgery	15.45 (23.54)	22.67 (28.59)	< 0.05*	14.17
6‐month post‐surgery	13.78 (23.95)	16.81 (24.61)	0.40	20.42
9‐month post‐surgery	8.92 (20.28)	21.10 (28.92)	< 0.05*	25.83
12‐month post‐surgery	13.89 (23.57)	14.60 (22.13)	0.84	27.08
*Appetite loss*				
Baseline	12.09 (25.59)	10.51 (21.60)	0.63	—
1‐month post‐surgery	22.89 (28.95)	11.44 (21.67)	< 0.05*	10.00
3‐month post‐surgery	12.20 (22.54)	11.73 (22.50)	0.89	14.17
6‐month post‐surgery	8.89 (20.01)	10.54 (22.17)	0.59	20.42
9‐month post‐surgery	4.69 (16.23)	8.56 (20.49)	0.16	25.83
12‐month post‐surgery	7.41 (17.89)	5.40 (15.42)	0.44	27.08

Abbreviations: EORTC QLQ‐C30, European Organisation for Research and Treatment of Cancer Core Quality of Life Questionnaire; GQOL, Global Health Status; SD, standard deviation.

* Denotes statistical significance.

The stoma group experienced significantly more severe fatigue (39.36 vs. 23.32, *p* < 0.05), nausea and vomiting (6.83 vs. 2.49, *p* < 0.05), pain (25.50 vs. 15.17, *p* < 0.05), dyspnoea (11.24 vs. 5.72, *p* < 0.05), insomnia (33.73 vs. 16.04, *p* < 0.05), and appetite loss (22.89 vs. 11.44, *p* < 0.05) at the 1‐month post‐surgery timepoint. Despite these statistical differences, only fatigue and appetite loss scores were clinically significant mainly because MIDs were not established for the other symptom scales. Insomnia scores were significantly higher for the no stoma group than the stoma group at the 3‐month (22.67 vs. 15.45, *p* < 0.05) and 9‐month (21.10 vs. 8.92, *p* < 0.05).

### 3.3 Baseline Predictors of HRQOL Scores Over Time

All multilevel mixed effect tobit models were statistically significant and explained the variance in their respective EORTC QLQ‐C30 outcome scores (Table 2). However, stoma creation was only significantly associated with poorer physical (*β* = −10.39, 95% CI: −15.80, −4.97) and role functioning (*β* = −12.24, 95% CI: −24.38, −0.10). Higher LOS significantly predicted poorer physical (*β* = −0.55, 95% CI: −0.94, −0.16) and role (*β* = −0.71, 95% CI: −1.37, −0.05) functioning, as well as higher fatigue (*β* = 0.40, 95% CI: 0.15, 0.65) and financial difficulties (*β* = 0.81, 95% CI: 0.25, 1.37), over time. Higher age at diagnosis was associated with higher GQOL (*β* = 0.25, 95% CI: 0.10, 0.39) and social functioning (β = 0.99, 95% CI: 0.33, 1.65), as well as lower nausea and vomiting (*β* = −0.11, 95% CI: −0.18, −0.03). Being male was associated with higher financial difficulties (*β* = 6.91, 95% CI: 0.97, 12.86) and appetite loss (*β* = 3.69, 95% CI: 0.24, 7.14). Lastly, receiving open surgery was associated with lower appetite loss (*β* = −4.12, 95% CI: −8.03, −0.22). Baseline scores were significantly associated with change in their respective HRQOL outcome scores over time in all models except for the nausea and vomiting scale.

**TABLE 2:** Model indices and fixed effect predictors for multilevel mixed effect tobit regression.

	*β*	Robust SE	*p*
*GQOL*	Wald *χ* ^2^ = 32.54		
Stoma creation (No stoma as reference group)	−2.00	2.15	0.35
GQOL baseline score	0.26	0.67	< 0.05*
CCI	0.08	0.10	0.41
Surgery type (Laparoscopic as reference group)	2.68	2.38	0.26
LOS	−0.33	0.18	0.07
Marital status (Married as reference group)	0.84	2.58	0.74
Age	0.25	0.07	< 0.05*
*Physical functioning*	Wald *χ* ^2^ = 80.16		
Stoma creation (No stoma as reference group)	−10.39	2.76	< 0.05*
Physical functioning baseline score	0.61	0.11	< 0.05*
CCI	0.17	0.13	0.21
Surgery type (Laparoscopic as reference group)	−1.95	3.15	0.54
LOS	−0.55	0.20	< 0.05*
Marital status (Married as reference group)	−2.52	3.31	0.45
*Role functioning*	Wald *χ* ^2^ = 34.87		
Stoma creation (No stoma as reference group)	−12.24	6.19	< 0.05*
Role functioning baseline score	0.63	0.15	< 0.05*
CCI	0.25	0.29	0.40
Surgery type (Laparoscopic as reference group)	2.35	7.69	0.76
LOS	−0.71	0.34	< 0.05*
Marital status (Married as reference group)	−1.69	8.01	0.83
*Emotional functioning*	Wald *χ* ^2^ = 31.98		
Stoma creation (No stoma as reference group)	−4.86	3.95	0.22
Emotional functioning baseline score	0.49	0.09	< 0.05*
CCI	0.16	0.14	0.28
Surgery type (Laparoscopic as reference group)	1.09	4.85	0.82
Marital status (Married as reference group)	−4.64	4.75	0.33
*Cognitive functioning*	Wald *χ* ^2^ = 26.45		
Stoma creation (No stoma as reference group)	11.37	7.70	0.14
Cognitive functioning baseline score	1.01	0.22	< 0.05*
CCI	−0.16	0.20	0.94
Surgery type (Laparoscopic as reference group)	−1.63	7.36	0.82
Tumour site (Colon as reference group)	−14.66	7.66	0.06
Marital status (Married as reference group)	−3.64	7.31	0.62
*Social functioning*	Wald *χ* ^2^ = 35.49		
Stoma creation (No stoma as reference group)	−1.38	7.85	0.86
Social functioning baseline score	0.71	0.18	< 0.05*
CCI	−0.12	0.33	0.72
Surgery type (Laparoscopic as reference group)	3.23	10.00	0.75
Age	0.99	0.34	< 0.05*
Marital status (Married as reference group)	−2.68	9.09	0.77
Sex (Male as reference group)	−12.17	7.48	0.10
*Fatigue*	Wald *χ* ^2^ = 60.92		
Stoma creation (No stoma as reference group)	1.21	2.26	0.59
Fatigue baseline score	0.27	0.07	< 0.05*
CCI	−0.09	0.10	0.32
Surgery type (Laparoscopic as reference group)	−0.79	2.88	0.78
LOS	0.40	0.13	< 0.05*
Marital status (Married as reference group)	−0.83	3.07	0.79
*Nausea and vomiting*	Wald *χ* ^2^ = 17.20		
Stoma creation (No stoma as reference group)	−0.59	0.97	0.54
Nausea and vomiting baseline score	0.10	0.06	0.11
CCI	0.03	0.05	0.57
Surgery type (Laparoscopic as reference group)	−0.98	0.94	0.30
Underwent radiotherapy (No radiotherapy as reference group)	2.71	1.70	0.11
Marital status (Married as reference group)	0.05	1.13	0.97
Age	−0.11	0.04	< 0.05*
*Pain*	Wald *χ* ^2^ = 41.23		
Stoma creation (No stoma as reference group)	0.98	2.03	0.63
Pain baseline score	0.23	0.05	< 0.05*
CCI	−0.01	0.05	0.96
Surgery type (Laparoscopic as reference group)	0.42	2.12	0.84
Tumour site (Colon as reference group)	3.94	2.14	0.07
Marital status (Married as reference group)	3.68	2.49	0.14
Age	−0.15	0.08	0.07
*Financial difficulties*	Wald *χ* ^2^ = 36.77		
Stoma creation (No stoma as reference group)	−4.63	5.07	0.36
Financial difficulties baseline score	0.38	0.08	< 0.05*
CCI	−0.31	0.18	0.09
Surgery type (Laparoscopic as reference group)	3.74	5.36	0.49
LOS	0.81	0.29	< 0.05*
Tumour site (Colon as reference group)	7.31	5.02	0.15
Marital status (Married as reference group)	2.34	4.87	0.63
Sex (Male as reference group)	6.91	3.03	< 0.05*
*Dyspnoea*	Wald *χ* ^2^ = 26.48		
Stoma creation (No stoma as reference group)	0.76	1.49	0.61
Dyspnoea baseline score	0.25	0.05	< 0.05*
CCI	< 0.01	0.05	0.99
Surgery type (Laparoscopic as reference group)	−1.27	1.03	0.51
Marital status (Married as reference group)	−1.26	1.70	0.46
*Insomnia*	Wald *χ* ^2^ = 19.42		
Stoma creation (No stoma as reference group)	−2.71	2.57	0.29
Insomnia baseline score	0.25	0.06	< 0.05*
CCI	−0.12	0.08	0.13
Surgery type (Laparoscopic as reference group)	0.27	2.98	0.93
Marital status (Married as reference group)	0.89	3.52	0.80
*Appetite loss*	Wald *χ* ^2^ = 24.56		
Stoma creation (No stoma as reference group)	−0.88	1.92	0.65
Appetite loss baseline score	0.19	0.06	< 0.05*
CCI	0.09	0.08	0.26
Surgery type (Laparoscopic as reference group)	−4.12	1.99	< 0.05*
Marital status (Married as reference group)	1.96	2.56	0.44
Sex (Male as reference group)	3.69	1.76	< 0.05*

Abbreviations: CCI, comprehensive complication index; GQOL, Global Health Status; LOS, length of stay; SE, standard error.

* Denotes statistical significance.

### 3.4 Sensitivity Analysis of Tobit Models With Permanent Ostomies Excluded

Excluding patients with permanent stomas did not significantly change associations between predictors and HRQOL outcome scores over time. The exception was the model for role functioning in which stoma creation was no longer statistically significant (*β* = −11.55, 95% CI: −24.77, 1.67), although the change in the point estimate was marginal.

### 3.5 Differences in HRQOL Between Ileostomy and Colostomy Patients

Baseline clinical and demographic characteristics for the ileostomy and colostomy subgroups were comparable except for stoma reversal—there were significantly more colostomy patients who required permanent stomas (*N* = 13, 35.1%) (Table S2). Table S3 presents bivariate comparisons in HRQOL outcome scores at each timepoint between the two subgroups. Between‐group HRQOL scores were relatively similar across the study period. The ileostomy subgroup was observed to consistently experience more severe financial difficulties than the colostomy subgroup, although these differences were not statistically significant.

### 3.6 Differences in HRQOL Between Temporary and Permanent Stoma Patients

Similarly, baseline clinical and demographic characteristics for the temporary and permanent stoma subgroups were comparable (Table S4). Table S5 presents bivariate comparisons in HRQOL outcome scores at each timepoint for the two subgroups. There were few statistically significant consistent between‐group differences, although the permanent stoma subgroup was observed to consistently experience poorer scores across all time points for cognitive functioning, with the greatest difference being at 1 month (73.08 vs. 91.19, *p* < 0.025). Descriptively, the permanent stoma subgroup tended to have poorer functioning scores and more severe symptom scores respectively at most timepoints across the study period.

## 4 Discussion

The present research aimed to assess HRQOL among CRC patients by comparing those with and without stomas and examining differences between subgroups. Stoma patients reported significantly poorer HRQOL, particularly in global health status and functional domains at 1 month post‐surgery, with physical functioning impairments persisting up to 12 months. Stoma creation predicted reduced physical and role functioning. Patients with permanent stomas consistently reported lower cognitive functioning scores than those with temporary stomas. Ileostomy patients experienced greater financial difficulties compared to colostomy patients, although this difference was not statistically significant.

Bivariate comparisons showed that stoma patients experienced worse HRQOL early in the treatment journey compared to those who did not require a stoma, especially at the 1‐month timepoint. This is supported by existing literature that the immediate post‐operative phase is accompanied by significant lifestyle adaptation for patients who undergo ostomy [8, 18, 19]. For example, He and colleagues qualitatively highlighted that ostomy patients in particular faced challenges relating to self‐acceptance, social isolation, as well as work‐ and personal‐life compromises [20].

Our longitudinal analyses also showed that stoma creation was a predictor of poorer physical and role functioning scores over time. This association was maintained for physical functioning even when patients with permanent stomas were removed as part of sensitivity analyses. Existing longitudinal studies have also yielded similar findings. For example, Herrle and colleagues found that patients with stoma creation experienced significant reductions in role and social functioning as well as gastrointestinal symptoms, with only partial recovery over time following stoma reversal [21]. Moving forward from a clinical perspective, post‐operative support for patients who underwent ostomy should not just focus on stoma and wound care but also help identify challenges in intra‐ and interpersonal life domains where the patient may be suffering. Patients who can care for their stoma competently should not simply be assumed to be coping well, and physiotherapists, social workers and community nurses likely play a crucial role in helping stoma patients adjust to post‐operative living [22, 23]. Evidence from the existing literature has demonstrated the efficacy of structured self‐management programs delivered via telehealth, enhanced follow‐up support provided by multidisciplinary stoma teams, and tailored interventions addressing issues related to body image, anxiety, and social isolation in improving HRQOL and patient self‐efficacy [24, 25, 26, 27]. Furthermore, dyadic interventions have been shown to reduce caregiver burden and foster healthy dyadic coping between patients and caregivers, emphasizing the importance of incorporating family support in the post‐operative care of patients with stomas [25, 28]. From a separate hypothetical viewpoint, this raises the question and further impetus to determine if early closure of a temporary stoma would result in faster recovery of physical and role functioning scores compared to delayed closure in suitable patients. Other longitudinal studies have reported no significant differences in HRQOL scores between early and late closure groups at 12 months [29, 30]. These findings align with ours, whereby impairments in HRQOL scores tend to persist beyond the immediate post‐operative period, regardless of stoma reversal timing. The decision to perform early versus late closure continues with proponents on either side of the clinical argument [31, 32]. Whether early closure of temporary stomas would in fact lead to superior quality of life scores remains controversial due to the purported higher peri‐operative complications that have been reported [33].

One interesting finding was that the ileostomy subgroup experienced more financial difficulties and appetite loss than the colostomy subgroup. Although it is beyond the scope of this study to conclude, one postulation is that patients with ileostomy may utilize stoma‐related appliances and cleansing materials at a higher frequency due to the relatively liquid nature of their enteric output [34]. Stoma‐related appliances (e.g., stoma pouches, elastic tape) can be costly and are often funded out‐of‐pocket rather than via insurance coverage in our local context. Some health systems have implemented subsidy‐ or co‐payment schemes to mitigate this burden. For example, the Australian government's Department of Health, Disability and Ageing has a Stoma Appliance scheme that acts as a sort of group insurance—enrollees enjoy completely subsidized stoma supplies which are covered by an annual access fee [35]. In addition, patients with ileostomy have been observed to experience higher rates of readmission due to dehydration from excessive ileostomy output or functional obstruction due to the ileostomy [36]. However, most colorectal surgeons are more comfortable performing a closure of ileostomy if the stoma is meant to be temporary and the literature remains divided on whether a colostomy or ileostomy is more ideal—especially after rectal cancer surgery. Nonetheless, we caveat that the subgroup analyses in this study were exploratory due to limited statistical power. Future studies seeking to draw more conclusive interpretations should consider strategies such as supersampling of patients with specific stoma characteristics to help ensure adequate sample sizes.

Regardless, patients who require stoma are likely to also be more complex, as suggested by the higher CCI and LOS of the stoma group in the present study. While there should be a continued focus on how to better improve the HRQOL of CRC patients who have undergone ostomy, we hope that our findings also urge colorectal surgeons and oncologists to consider upstream initiatives to prevent ostomy creation in the first place. Two relevant strategies to achieve these are the promotion of population‐level early detection for CRC (such as Singapore's Screen For Life for all individuals aged 50 years and above) and primary organ‐sparing treatment [37, 38]. In the case of the latter, recent evidence has started to highlight the role of neoadjuvant chemo‐radiotherapy from a HRQOL and organ preservation/functional perspective [38].

In summary, it is likely that interpersonal, cultural and social norms are all highly contextual factors influencing HRQOL in any stoma patient across the treatment journey, and that these may differ from population to population. One complementary area of work that should be considered is the reinforcing of self‐care in stoma patients. Recent research has suggested that such interventions may significantly improve HRQOL in stoma patients within the short term, and future studies could examine whether these enhancements persist over a longer observational period [39, 40, 41]. On a related note, several studies have also emphasized the importance of the spousal relationship in stoma patients' self‐acceptance of their ostomies, especially in managing the feeling of being “different” [42, 43, 44, 45]. The dyadic journey of both the patient (with a temporary or permanent stoma) and their spouse in adapting to this treatment paradigm is an under‐studied area that should be further considered.

### 4.1 Limitations

While our study is derived from a prospective, multi‐site cohort of CRC patients recruited from diagnosis and followed up until 12 months after surgery, there are two limitations to caveat. Firstly, the prospective sample was not initially designed to focus specifically on patients with stoma. This has yielded both strengths and limitations. On the one hand, the distribution of stoma versus non‐stoma patients is likely to be representative of the study sites' CRC population. However, this also meant that we had insufficient statistical power to perform more in‐depth longitudinal subgroup analyses. Secondly and common to most longitudinal studies, we experienced some losses to follow‐up across the study period. Loss to follow‐up can introduce biases into clinical studies as participants with poorer outcomes are more likely to drop out [46]. It is possible that attrition bias from our missing data could have partly explained both the high overall HRQOL in the sample as well as the relatively small differences in HRQOL outcome scores between subgroups. Lastly, we caution that there may be residual confounding from baseline characteristics not accounted for due to the non‐randomised design of this study.

### 4.2 Conclusions

Nonetheless, this study represents one of the first multisite longitudinal prospective comparisons of HRQOL in CRC patients, focusing specifically on the impact of ostomy creation, in Southeast Asia. Our findings broadly confirm that stoma patients experience poorer HRQOL especially in the earlier stages of the treatment journey. But more importantly, this study has identified potential gaps in our understanding of how the type of stoma created may have an impact on patients' lived experiences. Inroads should be made into tailoring post‐operative stoma support not just to help patients care for their stoma, but also to examine and address areas of daily living that have been unduly impacted.

## Author Contributions

**Jerrald Lau:** conceptualization (equal), formal analysis (equal), methodology (equal), validation (equal), writing – original draft (equal), writing – review and editing (equal). **Alyssa Ng:** data curation (equal), formal analysis (equal), investigation (equal), project administration (equal), validation (equal), writing – review and editing (equal). **Wei‐Ling Koh:** investigation (equal), project administration (equal), validation (equal), writing – review and editing (equal). **Cherie Hui Peh:** investigation (equal), project administration (equal), validation (equal), writing – review and editing (equal). **The Singapore Colorectal Cancer Research Group:** funding acquisition (equal), resources (equal), writing – review and editing (equal). **Nan Luo:** conceptualization (equal), supervision (equal), writing – review and editing (equal). **Ker‐Kan Tan:** conceptualization (equal), funding acquisition (equal), methodology (equal), resources (equal), supervision (equal), writing – review and editing (equal).

## Funding

This work was supported by the Singapore National Medical Research Council's Clinician Scientist Award (MOH‐000333‐00) and Health Services Research Grant (MOH‐000743). The funders had no role in the conceptualisation, design, operationalisation of the study and analysis of the study's findings.

## Ethics Statement

Approval to proceed with this study was provided by the National Healthcare Group's Domain Specific Review Board (NHG DSRB Ref: 2017/00518) in accordance with the Declaration of Helsinki. Written informed consent was obtained from all patients participating in this study.

## Conflicts of Interest

The authors declare no conflicts of interest.

## Supporting information


**Table S1:** Baseline sociodemographic and clinical descriptives of CRC patients with and without stoma.
**Table S2:** Baseline sociodemographic and clinical descriptives of the ileostomy and colostomy subgroups.
**Table S3:** EORTC QLQ‐C30 scores between ileostomy and colostomy subgroups across the study period.
**Table S4:** Baseline sociodemographic and clinical descriptives of the temporary and permanent stoma subgroups.
**Table S5:** EORTC QLQ‐C30 scores between temporary and permanent stoma subgroups across the study period.

## Data Availability

The data that support the findings of this study are available on request from the corresponding author, KKT. The data are not publicly available due to potentially identifiable information derived from participants' electronic medical records.
