# Supplementary material for: Associations Between Ostomy Creation and Health‐Related Quality of Life in Colorectal Cancer Patients: A Longitudinal Observational Study
Source: Cancer Med. 2025 Nov 28;14(23):e71388. doi: 10.1002/cam4.71388 (PMC12661462; doi:10.1002/cam4.71388)
Supplement: Supplementary file 1 — Table S1: Baseline sociodemographic and clinical descriptives of CRC patients with and without stoma. Table S2: Baseline sociodemographic and clinical descriptives of the ileostomy and colostomy subgroups. Table S3: EORTC QLQ‐C30 scores between ileostomy and colostomy subgroups across the study period. Table S4: Baseline sociodemographic and clinical descriptives of the temporary and permanent stoma subgroups. Table S5: EORTC QLQ‐C30 scores between temporary and permanent stoma subgroups across the study period. [file CAM4-14-e71388-s001.docx]

**Supplementary** **Table 1.** Baseline sociodemographic and clinical descriptives of CRC patients with and without stoma.

|  | Stoma  (N = 91) | No stoma  (N = 149) | p-value |
| --- | --- | --- | --- |
| Age, mean years (SD) | 67.09 (12.09) | 66.54 (11.11) | 0.72 |
| Race, N (%) |  |  |  |
| Chinese | 78 (85.71) | 123 (82.55) |  |
| Malay | 10 (10.99) | 19 (12.75) |  |
| Indian | 3 (3.30) | 4 (2.68) |  |
| Other | 0 (0.00) | 3 (2.01) | 0.67 |
| Sex, N (%) |  |  |  |
| Female | 34 (37.36) | 65 (43.62) |  |
| Male | 57 (62.64) | 84 (56.38) | 0.34 |
| Marital status, N (%) |  |  |  |
| Married | 63 (81.82) | 94 (81.03) |  |
| Unmarried, divorced or widowed | 14 (18.18) | 22 (18.97) | 0.89 |
| Tumour site, N (%) |  |  |  |
| Colon | 33 (36.26) | 129 (86.58) |  |
| Rectum | 58 (63.74) | 20 (13.42) | <0.05* |
| Type of surgery, N (%) |  |  |  |
| Laparoscopic | 53 (71.62) | 98 (84.48) |  |
| Open | 21 (28.38) | 18 (15.52) | <0.05* |
| Stoma type, N (%) |  |  |  |
| Ileostomy | 54 (59.34) | - |  |
| Colostomy | 37 (40.66) | - |  |
| Stoma reversal, N (%) |  |  |  |
| Temporary | 76 (83.52) | - |  |
| Permanent | 15 (16.48) | - |  |
| Cancer stage, N (%) |  |  |  |
| I | 15 (17.65) | 24 (17.65) |  |
| II | 36 (42.35) | 46 (33.82) |  |
| III | 25 (29.41) | 55 (40.44) |  |
| IV | 9 (10.59) | 11 (8.09) | 0.37 |
| Underwent chemotherapy, N (%) |  |  |  |
| No | 35 (39.33) | 63 (44.68) |  |
| Yes | 54 (60.67) | 78 (55.32) | 0.42 |
| Underwent radiotherapy, N (%) |  |  |  |
| No | 57 (64.04) | 139 (97.20) |  |
| Yes | 32 (35.96) | 4 (2.80) | <0.05* |
| ASA classification, N (%) |  |  |  |
| I | 1 (1.32) | 5 (4.27) |  |
| II | 57 (75.00) | 93 (79.49) |  |
| III | 17 (22.37) | 19 (16.24) |  |
| IV | 1 (1.32) | 0 (0.00) | 0.27 |
| CCI, mean score (SD) | 11.23 (15.81) | 5.90 (10.21) | <0.05* |
| Total LOS, mean days (SD) | 11.05 (10.57) | 6.86 (3.43) | <0.05* |
| Readmission within 30 days post-surgery, N (%) |  |  |  |
| No | 76 (87.36) | 128 (94.81) |  |
| Yes | 11 (12.64) | 7 (5.19) | 0.05 |

Note: * denotes statistical significance. The following abbreviations were used - colorectal cancer (CRC); standard deviation (SD); American Society of Anesthesiologists (ASA); comprehensive complication index (CCI); length of stay (LOS)

**Supplementary Table 2.** Baseline sociodemographic and clinical descriptives of the ileostomy and colostomy subgroups.

|  | Ileostomy  (N = 54) | Colostomy  (N = 37) | p-value |
| --- | --- | --- | --- |
| Age, mean years (SD) | 65.96 (11.25) | 68.73 (13.22) | 0.30 |
| Race, N (%) |  |  |  |
| Chinese | 45 (83.33) | 33 (89.19) |  |
| Malay | 7 (12.96) | 3 (8.11) |  |
| Indian | 2 (3.70) | 1 (2.70) |  |
| Other | 0 (0.00) | 0 (0.00) | 0.79 |
| Sex, N (%) |  |  |  |
| Female | 18 (33.33) | 16 (43.24) |  |
| Male | 36 (66.67) | 21 (56.76) | 0.34 |
| Marital status, N (%) |  |  |  |
| Married | 38 (82.61) | 25 (80.65) |  |
| Unmarried, divorced or widowed | 8 (17.39) | 6 (19.35) | 0.83 |
| Tumour site, N (%) |  |  |  |
| Colon | 17 (31.48) | 16 (43.24) |  |
| Rectum | 37 (68.52) | 21 (56.76) | 0.25 |
| Type of surgery, N (%) |  |  |  |
| Laparoscopic | 34 (73.91) | 19 (67.86) |  |
| Open | 12 (26.09) | 9 (32.14) | 0.58 |
| Stoma reversal, N (%) |  |  |  |
| Temporary | 52 (96.30) | 24 (64.86) |  |
| Permanent | 2 (3.70) | 13 (35.14) | <0.05* |
| Cancer stage, N (%) |  |  |  |
| I | 10 (19.23) | 5 (15.15) |  |
| II | 21 (40.38) | 15 (45.45) |  |
| III | 17 (32.69) | 8 (24.24) |  |
| IV | 4 (7.69) | 5 (15.15) | 0.62 |
| Underwent chemotherapy, N (%) |  |  |  |
| No | 21 (38.89) | 14 (40.00) |  |
| Yes | 33 (61.11) | 21 (60.00) | 0.92 |
| Underwent radiotherapy, N (%) |  |  |  |
| No | 33 (61.11) | 24 (68.57) |  |
| Yes | 21 (38.89) | 11 (31.43) | 0.47 |
| ASA classification, N (%) |  |  |  |
| I | 1 (2.17) | 0 (0.00) |  |
| II | 36 (78.26) | 21 (70.00) |  |
| III | 9 (19.57) | 8 (26.67) |  |
| IV | 0 (0.00) | 1 (3.33) | 0.53 |
| CCI, mean score (SD) | 8.30 (12.16) | 15.69 (19.52) | 0.08 |
| Total LOS, mean days (SD) | 9.10 (4.09) | 14.03 (15.63) | 0.10 |
| Readmission within 30 days post-surgery, N (%) |  |  |  |
| No | 48 (88.89) | 28 (84.85) |  |
| Yes | 6 (11.11) | 5 (15.15) | 0.74 |

Note: * denotes statistical significance. The following abbreviations were used - standard deviation (SD); American Society of Anesthesiologists (ASA); comprehensive complication index (CCI); length of stay (LOS)

**Supplementary Table 3.** EORTC QLQ-C30 scores between ileostomy and colostomy subgroups across the study period.

| GQOL and functioning scales, mean (SD) | Ileostomy  (N = 54) | Colostomy  (N = 37) | p-value† |
| --- | --- | --- | --- |
| Global health status |  |  |  |
| Baseline | 68.36 (20.05) | 64.41 (20.57) | 0.36 |
| 1-month post-surgery | 59.97 (20.35) | 63.28 (19.84) | 0.47 |
| 3-month post-surgery | 67.88 (19.14) | 67.68 (20.17) | 0.96 |
| 6-month post-surgery | 74.28 (17.99) | 68.39 (22.86) | 0.22 |
| 9-month post-surgery | 71.63 (16.87) | 74.14 (15.49) | 0.53 |
| 12-month post-surgery | 71.78 (16.98) | 72.62 (20.14) | 0.85 |
| Physical functioning |  |  |  |
| Baseline | 92.58 (12.99) | 83.42 (23.23) | 0.03 |
| 1-month post-surgery | 70.80 (24.39) | 62.58 (26.87) | 0.16 |
| 3-month post-surgery | 80.14 (20.48) | 79.39 (25.35) | 0.88 |
| 6-month post-surgery | 86.96 (19.27) | 83.91 (18.73) | 0.50 |
| 9-month post-surgery | 84.13 (21.28) | 84.37 (18.71) | 0.96 |
| 12-month post-surgery | 89.61 (17.60) | 83.33 (18.21) | 0.16 |
| Role functioning |  |  |  |
| Baseline | 93.83 (13.85) | 86.94 (25.20) | 0.14 |
| 1-month post-surgery | 59.80 (34.51) | 60.42 (35.10) | 0.94 |
| 3-month post-surgery | 74.49 (32.29) | 81.82 (27.12) | 0.29 |
| 6-month post-surgery | 85.14 (26.11) | 87.36 (19.75) | 0.70 |
| 9-month post-surgery | 88.89 (22.59) | 88.51 (21.41) | 0.94 |
| 12-month post-surgery | 91.29 (17.79) | 86.90 (24.58) | 0.38 |
| Emotional functioning |  |  |  |
| Baseline | 79.17 (19.41) | 80.86 (17.55) | 0.67 |
| 1-month post-surgery | 73.86 (27.89) | 81.25 (21.89) | 0.21 |
| 3-month post-surgery | 81.80 (21.43) | 82.58 (20.87) | 0.87 |
| 6-month post-surgery | 87.86 (18.57) | 87.36 (17.90) | 0.91 |
| 9-month post-surgery | 92.26 (11.55) | 86.78 (18.70) | 0.13 |
| 12-month post-surgery | 90.15 (12.99) | 88.99 (14.35) | 0.72 |
| Cognitive functioning |  |  |  |
| Baseline | 95.68 (10.33) | 92.79 (12.75) | 0.24 |
| 1-month post-surgery | 91.18 (18.06) | 83.85 (23.75) | 0.12 |
| 3-month post-surgery | 93.88 (12.59) | 92.93 (13.20) | 0.74 |
| 6-month post-surgery | 93.12 (13.41) | 95.40 (10.82) | 0.44 |
| 9-month post-surgery | 94.84 (11.34) | 91.95 (15.82) | 0.37 |
| 12-month post-surgery | 93.41 (13.68) | 89.88 (14.59) | 0.30 |
| Social functioning |  |  |  |
| Baseline | 87.65 (22.47) | 87.39 (22.70) | 0.96 |
| 1-month post-surgery | 69.28 (34.86) | 77.08 (31.89) | 0.31 |
| 3-month post-surgery | 81.29 (28.59) | 87.88 (17.81) | 0.24 |
| 6-month post-surgery | 88.04 (21.85) | 87.36 (20.24) | 0.89 |
| 9-month post-surgery | 93.25 (16.49) | 91.95 (17.03) | 0.75 |
| 12-month post-surgery | 94.32 (15.64) | 89.29 (23.66) | 0.28 |
| Symptom scales (excluding constipation and diarrhoea), mean (SD) | Ileostomy  (N = 54) | Colostomy  (N = 37) | p-value |
| Fatigue |  |  |  |
| Baseline | 20.72 (19.97) | 18.31 (18.71) | 0.56 |
| 1-month post-surgery | 39.43 (26.97) | 39.24 (29.19) | 0.98 |
| 3-month post-surgery | 26.07 (21.58) | 22.90 (22.38) | 0.52 |
| 6-month post-surgery | 20.29 (21.12) | 21.84 (18.66) | 0.75 |
| 9-month post-surgery | 19.31 (18.04) | 19.92 (19.33) | 0.89 |
| 12-month post-surgery | 20.45 (20.74) | 21.43 (21.37) | 0.85 |
| Nausea and vomiting |  |  |  |
| Baseline | 1.23 (5.47) | 4.05 (17.31) | 0.27 |
| 1-month post-surgery | 8.17 (20.10) | 4.69 (12.15) | 0.38 |
| 3-month post-surgery | 4.42 (10.64) | 3.13 (7.85) | 0.56 |
| 6-month post-surgery | 2.90 (10.72) | 3.45 (9.32) | 0.82 |
| 9-month post-surgery | 1.98 (10.54) | 5.17 (19.47) | 0.38 |
| 12-month post-surgery | 4.17 (16.12) | 2.98 (10.20) | 0.73 |
| Pain |  |  |  |
| Baseline | 8.64 (16.43) | 12.61 (24.97) | 0.36 |
| 1-month post-surgery | 26.47 (29.09) | 23.96 (25.38) | 0.69 |
| 3-month post-surgery | 15.31 (21.74) | 12.63 (21.26) | 0.58 |
| 6-month post-surgery | 10.14 (20.33) | 8.62 (15.82) | 0.73 |
| 9-month post-surgery | 11.51 (20.66) | 12.07 (18.84) | 0.91 |
| 12-month post-surgery | 11.36 (20.58) | 12.5 (17.35) | 0.81 |
| Financial difficulties |  |  |  |
| Baseline | 22.84 (30.94) | 19.82 (36.40) | 0.67 |
| 1-month post-surgery | 24.18 (33.39) | 16.67 (32.79) | 0.32 |
| 3-month post-surgery | 21.09 (31.69) | 14.14 (25.04) | 0.29 |
| 6-month post-surgery | 21.01 (33.96) | 13.79 (31.52) | 0.36 |
| 9-month post-surgery | 16.67 (28.75) | 13.79 (30.23) | 0.69 |
| 12-month post-surgery | 18.18 (33.30) | 10.71 (25.75) | 0.32 |
| Dyspnoea |  |  |  |
| Baseline | 5.56 (14.11) | 6.31 (13.24) | 0.80 |
| 1-month post-surgery | 9.80 (20.32) | 13.54 (22.17) | 0.43 |
| 3-month post-surgery | 6.80 (17.98) | 7.07 (16.15) | 0.95 |
| 6-month post-surgery | 6.52 (18.08) | 4.60 (11.70) | 0.61 |
| 9-month post-surgery | 3.97 (10.93) | 11.49 (20.46) | 0.08 |
| 12-month post-surgery | 6.82 (18.44) | 11.90 (18.62) | 0.26 |
| Insomnia |  |  |  |
| Baseline | 20.37 (27.02) | 26.13 (35.25) | 0.38 |
| 1-month post-surgery | 32.03 (32.63) | 36.46 (40.04) | 0.58 |
| 3-month post-surgery | 17.69 (23.67) | 12.12 (23.30) | 0.30 |
| 6-month post-surgery | 13.77 (23.91) | 13.79 (24.43) | 0.10 |
| 9-month post-surgery | 9.52 (18.46) | 8.05 (22.98) | 0.77 |
| 12-month post-surgery | 12.88 (21.82) | 15.48 (26.42) | 0.65 |
| Appetite loss |  |  |  |
| Baseline | 8.02 (15.78) | 18.02 (34.78) | 0.07 |
| 1-month post-surgery | 24.18 (30.61) | 20.83 (26.44) | 0.61 |
| 3-month post-surgery | 14.97 (24.59) | 8.08 (18.69) | 0.18 |
| 6-month post-surgery | 10.87 (22.28) | 5.75 (15.61) | 0.28 |
| 9-month post-surgery | 3.17 (9.90) | 6.90 (22.50) | 0.35 |
| 12-month post-surgery | 6.82 (18.44) | 8.33 (17.27) | 0.73 |

Note: † critical value of p<0.025 based on Bonferroni correction; * denotes statistical significance. The following abbreviations were used - European Organisation for Research and Treatment of Cancer Core Quality of Life Questionnaire (EORTC QLQ-C30); Global Health Status (GQOL); standard deviation (SD)

**Supplementary Table 4.** Baseline sociodemographic and clinical descriptives of the temporary and permanent stoma subgroups.

|  | Temporary  (N = 76) | Permanent  (N = 15) | p-value |
| --- | --- | --- | --- |
| Age, mean years (SD) | 66.18 (12.20) | 71.67 (10.78) | 0.10 |
| Race, N (%) |  |  |  |
| Chinese | 64 (84.21) | 14 (93.33) |  |
| Malay | 9 (11.84) | 1 (6.67) |  |
| Indian | 3 (3.95) | 0 (0.00) |  |
| Other | 0 (0.00) | 0 (0.00) | 1.00 |
| Sex, N (%) |  |  |  |
| Female | 26 (34.21) | 8 (53.33) |  |
| Male | 50 (65.79) | 7 (46.67) | 0.24 |
| Marital status, N (%) |  |  |  |
| Married | 52 (83.87) | 11 (73.33) |  |
| Unmarried, divorced or widowed | 10 (16.13) | 4 (26.67) | 0.46 |
| Tumour site, N (%) |  |  |  |
| Colon | 30 (39.47) | 3 (20.00) |  |
| Rectum | 46 (60.53) | 12 (80.00) | 0.24 |
| Type of surgery, N (%) |  |  |  |
| Laparoscopic | 42 (70.00) | 11 (78.57) |  |
| Open | 18 (30.00) | 3 (21.43) | 0.74 |
| Stoma type, N (%) |  |  |  |
| Ileostomy | 52 (68.42) | 2 (13.33) |  |
| Colostomy | 24 (31.58) | 13 (86.67) | <0.05* |
| Cancer stage, N (%) |  |  |  |
| I | 12 (17.14) | 3 (20.00) |  |
| II | 30 (42.86) | 6 (40.00) |  |
| III | 23 (32.86) | 2 (13.33) |  |
| IV | 5 (7.14) | 4 (26.67) | 0.11 |
| Underwent chemotherapy, N (%) |  |  |  |
| No | 29 (39.19) | 6 (40.00) |  |
| Yes | 45 (60.81) | 9 (60.00) | 0.95 |
| Underwent radiotherapy, N (%) |  |  |  |
| No | 46 (62.16) | 11 (73.33) |  |
| Yes | 28 (37.84) | 4 (26.67) | 0.56 |
| ASA classification, N (%) |  |  |  |
| I | 1 (1.64) | 0 (0.00) |  |
| II | 46 (75.41) | 11 (73.33) |  |
| III | 14 (22.95) | 3 (20.00) |  |
| IV | 0 (0.00) | 1 (6.67) | 0.42 |
| CCI, mean score (SD) | 10.35 (15.55) | 14.97 (16.94) | 0.25 |
| Total LOS, mean days (SD) | 10.87 (10.95) | 11.80 (9.18) | 0.40 |
| Readmission within 30 days post-surgery, N (%) |  |  |  |
| No | 65 (90.28) | 11 (73.33) |  |
| Yes | 7 (9.72) | 4 (26.67) | 0.09 |

Note: * denotes statistical significance. The following abbreviations were used - standard deviation (SD); American Society of Anesthesiologists (ASA); comprehensive complication index (CCI); length of stay (LOS)

**Supplementary Table 5.** EORTC QLQ-C30 scores between temporary and permanent stoma subgroups across the study period.

| GQOL and functioning scales, mean (SD) | Temporary  (N = 76) | Permanent  (N = 15) | p-value† |
| --- | --- | --- | --- |
| Global health status |  |  |  |
| Baseline | 66.45 (20.23) | 68.33 (20.94) | 0.75 |
| 1-month post-surgery | 61.90 (20.39) | 57.69 (18.78) | 0.33 |
| 3-month post-surgery | 68.53 (19.45) | 64.29 (19.73) | 0.48 |
| 6-month post-surgery | 73.28 (17.97) | 65.28 (28.83) | 0.60 |
| 9-month post-surgery | 72.56 (16.22) | 73.08 (17.06) | 0.98 |
| 12-month post-surgery | 71.89 (18.11) | 73.08 (18.99) | 0.87 |
| Physical functioning |  |  |  |
| Baseline | 89.6 (17.92) | 84.89 (20.7) | 0.43 |
| 1-month post-surgery | 70.98 (23.49) | 50.26 (29.52) | 0.03 |
| 3-month post-surgery | 80.00 (23.15) | 79.05 (19.19) | 0.64 |
| 6-month post-surgery | 86.35 (19.73) | 82.78 (14.90) | 0.19 |
| 9-month post-surgery | 84.94 (21.21) | 81.03 (14.62) | 0.08 |
| 12-month post-surgery | 87.59 (19.04) | 85.13 (14.44) | 0.23 |
| Role functioning |  |  |  |
| Baseline | 91.45 (18.76) | 88.89 (23.29) | 0.73 |
| 1-month post-surgery | 62.14 (34.51) | 48.72 (33.65) | 0.19 |
| 3-month post-surgery | 78.19 (30.51) | 73.81 (30.46) | 0.49 |
| 6-month post-surgery | 87.30 (22.74) | 79.17 (28.54) | 0.40 |
| 9-month post-surgery | 89.94 (21.40) | 83.33 (24.53) | 0.21 |
| 12-month post-surgery | 90.39 (20.34) | 85.90 (22.41) | 0.34 |
| Emotional functioning |  |  |  |
| Baseline | 79.82 (18.67) | 80.00 (18.85) | 0.10 |
| 1-month post-surgery | 77.38 (26.59) | 73.08 (22.09) | 0.34 |
| 3-month post-surgery | 83.21 (21.28) | 76.79 (19.93) | 0.15 |
| 6-month post-surgery | 88.23 (17.30) | 84.72 (22.98) | 0.92 |
| 9-month post-surgery | 91.67 (11.58) | 82.69 (24.64) | 0.66 |
| 12-month post-surgery | 90.4 (12.65) | 86.54 (16.85) | 0.52 |
| Cognitive functioning |  |  |  |
| Baseline | 95.18 (11.49) | 91.11 (10.67) | 0.20 |
| 1-month post-surgery | 91.19 (19.40) | 73.08 (21.01) | 0.01* |
| 3-month post-surgery | 94.85 (11.61) | 86.90 (16.25) | 0.10 |
| 6-month post-surgery | 94.97 (11.84) | 88.89 (14.79) | 0.20 |
| 9-month post-surgery | 95.40 (11.59) | 85.90 (17.80) | 0.09 |
| 12-month post-surgery | 92.82 (13.29) | 88.46 (17.19) | 0.41 |
| Social functioning |  |  |  |
| Baseline | 89.69 (19.43) | 76.67 (32.61) | 0.15 |
| 1-month post-surgery | 74.76 (32.57) | 58.97 (38.26) | 0.17 |
| 3-month post-surgery | 85.78 (24.48) | 75.00 (25.94) | 0.17 |
| 6-month post-surgery | 87.83 (22.24) | 87.50 (14.43) | 0.32 |
| 9-month post-surgery | 94.54 (14.44) | 84.62 (23.04) | 0.08 |
| 12-month post-surgery | 92.66 (19.15) | 91.03 (19.97) | 0.75 |
| Symptom scales (excluding constipation and diarrhoea), mean (SD) | Temporary  (N = 76) | Permanent  (N = 15) | p-value |
| Fatigue |  |  |  |
| Baseline | 17.54 (17.99) | 28.15 (22.95) | 0.09 |
| 1-month post-surgery | 36.03 (26.38) | 57.26 (28.63) | 0.02* |
| 3-month post-surgery | 23.53 (21.51) | 30.95 (23.13) | 0.24 |
| 6-month post-surgery | 19.75 (20.59) | 26.85 (16.72) | 0.12 |
| 9-month post-surgery | 17.43 (17.92) | 29.06 (18.45) | 0.05 |
| 12-month post-surgery | 19.40 (21.10) | 27.35 (19.04) | 0.12 |
| Nausea and vomiting |  |  |  |
| Baseline | 2.63 (12.78) | 1.11 (4.30) | 1.00 |
| 1-month post-surgery | 6.43 (17.79) | 8.97 (16.12) | 0.32 |
| 3-month post-surgery | 4.23 (9.77) | 2.38 (8.91) | 0.58 |
| 6-month post-surgery | 2.91 (10.17) | 4.17 (10.36) | 0.64 |
| 9-month post-surgery | 1.44 (8.99) | 11.54 (28.37) | 0.12 |
| 12-month post-surgery | 3.11 (14.00) | 6.41 (14.50) | 0.22 |
| Pain |  |  |  |
| Baseline | 9.21 (17.72) | 15.56 (30.52) | 0.80 |
| 1-month post-surgery | 23.10 (26.66) | 38.46 (29.96) | 0.06 |
| 3-month post-surgery | 12.75 (20.79) | 21.43 (23.96) | 0.13 |
| 6-month post-surgery | 9.26 (18.40) | 11.11 (20.52) | 0.94 |
| 9-month post-surgery | 10.63 (19.92) | 16.67 (19.25) | 0.09 |
| 12-month post-surgery | 9.89 (18.60) | 20.51 (20.59) | 0.06 |
| Financial difficulties |  |  |  |
| Baseline | 20.61 (31.25) | 26.67 (42.16) | 0.88 |
| 1-month post-surgery | 19.05 (31.38) | 33.33 (40.82) | 0.25 |
| 3-month post-surgery | 16.18 (27.91) | 28.57 (34.24) | 0.11 |
| 6-month post-surgery | 17.99 (32.70) | 19.44 (36.12) | 1.00 |
| 9-month post-surgery | 13.22 (26.45) | 25.64 (38.86) | 0.26 |
| 12-month post-surgery | 14.12 (29.82) | 20.51 (34.80) | 0.52 |
| Dyspnoea |  |  |  |
| Baseline | 6.14 (14.10) | 4.44 (11.73) | 0.99 |
| 1-month post-surgery | 10.48 (20.88) | 15.38 (22.01) | 0.37 |
| 3-month post-surgery | 6.37 (16.55) | 9.52 (20.37) | 0.58 |
| 6-month post-surgery | 4.76 (14.49) | 11.11 (21.71) | 0.30 |
| 9-month post-surgery | 6.32 (14.59) | 10.26 (21.01) | 0.58 |
| 12-month post-surgery | 8.47 (19.17) | 10.26 (16.01) | 0.63 |
| Insomnia |  |  |  |
| Baseline | 19.30 (28.42) | 40 (36.08) | 0.05 |
| 1-month post-surgery | 29.05 (33.05) | 58.97 (38.86) | 0.02* |
| 3-month post-surgery | 14.22 (22.54) | 21.43 (28.06) | 0.30 |
| 6-month post-surgery | 12.70 (23.52) | 19.44 (26.43) | 0.37 |
| 9-month post-surgery | 9.20 (21.44) | 7.69 (14.62) | 0.10 |
| 12-month post-surgery | 11.30 (20.16) | 25.64 (33.76) | 0.14 |
| Appetite loss |  |  |  |
| Baseline | 11.40 (24.07) | 15.56 (33.01) | 0.97 |
| 1-month post-surgery | 21.90 (29.43) | 28.21 (26.69) | 0.34 |
| 3-month post-surgery | 12.25 (22.97) | 11.90 (21.11) | 1.00 |
| 6-month post-surgery | 7.41 (19.35) | 16.67 (22.47) | 0.10 |
| 9-month post-surgery | 4.02 (12.61) | 7.69 (27.74) | 0.87 |
| 12-month post-surgery | 6.78 (17.26) | 10.26 (21.01) | 0.57 |

Note: † critical value of p<0.025 based on Bonferroni correction; * denotes statistical significance. The following abbreviations were used - European Organisation for Research and Treatment of Cancer Core Quality of Life Questionnaire (EORTC QLQ-C30); Global Health Status (GQOL); standard deviation (SD)
